# Comparison of Fetal Middle Cerebral Artery and Umbilical Artery Doppler Indices as Predictors of Perinatal Outcomes in Fetal Growth-Restricted Pregnancies

**DOI:** 10.7759/cureus.87102

**Published:** 2025-07-01

**Authors:** Priya Singh, Sonal Prasad, Natasha Tyagi, Richa Dabas, Ravi P Jha

**Affiliations:** 1 Obstetrics and Gynecology, Dr. Baba Saheb Ambedkar Medical College and Hospital, New Delhi, IND; 2 Community Medicine, Dr. Baba Saheb Ambedkar Medical College and Hospital, New Delhi, IND

**Keywords:** fetal growth restriction (fgr), middle cerebral artery pulsatility index (mca pi), nicu admissions, small for gestational age (sga), umbilical artery pulsatility index (ua pi), umbilical artery systolic diastolic ratio (uas/d)

## Abstract

Introduction: Fetal growth restriction (FGR) is considered a major contributor to perinatal morbidity and mortality. Umbilical artery (UA) Doppler has been widely utilized for assessing FGR. Recent evidence suggests that Doppler assessment of the middle cerebral artery (MCA) may also serve as a valuable tool in predicting perinatal outcomes.

Objectives: The objective of this study is to compare the predictive value of Doppler indices, specifically the MCA pulsatility index (PI), UA PI, and systolic/diastolic (S/D) ratio, in determining adverse perinatal outcomes in pregnancies complicated by FGR.

Methodology: This prospective observational study was conducted on 91 pregnant women with clinically and sonographically diagnosed FGR in the third trimester at a tertiary care center in Delhi. Post-delivery, neonates were categorized into small for gestational age (SGA) and non-SGA groups. Doppler parameters, including UA PI, UA S/D ratio, and MCA PI, along with variables such as age, parity, body mass index (BMI), and neonatal outcomes, were compared between the SGA and non-SGA groups.

Results: Among the participants after delivery, 69 mothers had SGA while 22 mothers had non-SGA neonates. MCA PI and UA S/D ratio showed statistically significant differences between the SGA and non-SGA groups (*P *= 0.017 and *P *= 0.023, respectively), while UA PI did not show a significant difference (*P *= 0.57). Abnormal UA S/D ratio were associated with higher rates of cesarean delivery when compared with normal doppler indices (27, 71.05%, vs. 20, 40.82%, *P* = 0.005), birth weight <10th percentile (33, 86.84%, vs. 32, 65.31%, *P* = 0.022), Neonatal Intensive Care Unit (NICU) admissions (29, 78.38%, vs. 22, 44.90%, *P* = 0.002), and neonatal complications (25, 65.79%, vs. 19, 38.78%, *P* = 0.012). Abnormal UA PI was linked to higher rates of neonatal complications (68.18% vs. 43.94%, *P *= 0.04). Abnormal MCA PI showed a significant association with birth weight <10th percentile (89.66% vs. 69.35%, *P* = 0.039). UA S/D demonstrated higher sensitivity (50.62%) and diagnostic accuracy (54.95%), whereas MCA PI showed higher specificity and positive predictive value (100% each) compared to UA PI.

Conclusions: This study's results indicated that abnormal UA S/D was significantly more pronounced than both MCA PI and UA PI. Sensitivity and diagnostic accuracy of UA S/D for SGA prediction were higher, specificity and predictive values of MCA PI were higher than those for UA PI. Thus, MCA PI Doppler indices were a better predictor for fetal outcome in FGR when compared with UA in terms of specificity and predictive value.

## Introduction

Fetal growth restriction (FGR) is a commonly encountered condition in obstetrics. FGR is a condition in which a fetus fails to achieve its growth potential and is consequently at risk of increased perinatal morbidity and mortality [1]. Such fetuses also suffer long-term complications such as increased risk of subsequent adult hypertension, atherosclerosis, type 2 diabetes, and metabolic derangement [2,3]. It is difficult to differentiate between suboptimal fetal growth due to intrauterine starvation and adequate growth of a constitutionally small infant. FGR is a common clinical sign of chronic fetal hypoxemia. Based on the time of onset, it is classified into two groups: early (before 32 weeks) and late (after 32 weeks) onset. Some factors leading to FGR include maternal causes (diabetes, hypertension, anemia, malnutrition, cardiopulmonary disease, smoking, drug use), fetal causes (genetic disease including congenital malformations, aneuploidy, fetal infection, multiple pregnancies), and placental causes (placental insufficiency, placental infarction, placental mosaicism) [4-7]. Doppler ultrasound [8] is a noninvasive method for the study of fetal hemodynamics and monitoring fetoplacental and uteroplacental circulation during pregnancy. It is safe in pregnancy and can be repeated when indicated. It can predict adverse perinatal outcomes and is considered a sensitive tool in the early detection of fetal compromise, enabling timely intervention. The Doppler patterns follow a longitudinal drift [9] with early changes in the middle cerebral artery (MCA) and umbilical artery (UA), followed by other peripheral arteries, which indicates redistribution of blood flow in growth-restricted fetuses and therefore cautions us to closely screen the fetus and intervene before the circumstances become unsalvageable. If adequate actions are not taken at this point, venous changes appear in the severely compromised fetus. These are strong predictors of poor perinatal outcome and indicate imminent irreversible damage. UA color Doppler has been subjected to more vigorous assessment than any previous test of fetal health. The UA differs from other vessels in that it normally has forward flow throughout the cardiac cycle. The amount of flow during diastole increases as gestation advances, and this reflects decreasing placental impedance. As a result, the systolic/diastolic (S/D) ratio normally declines from approximately 4.0 at 20 weeks gestation, to generally less than 3.0 after 30 weeks, and finally to 2.0 at term. The waveform is considered abnormal if the S/D ratio is >95th percentile for gestational age. In extreme cases of growth restriction, end-diastolic flow can become absent or even reversed. UA pulsatility index (PI) in the upper 95th percentile was considered an abnormal range (value more than 1.2) [10]. The MCA also predicts fetal outcome from alterations in cerebral blood flow and its direction. It was confirmed that during the prenatal period, the resistance in the cerebral artery of the fetus is high. However, this parameter can change in threatening conditions, such as placental insufficiency and hypoxemia, due to stimulation of chemoreceptors and alteration in vasodilator or vasoconstrictor agents. MCA Doppler [11] measurement has been advocated as an efficient modality for the detection of fetal hypoxia, which leads to perinatal adverse outcomes and fetal compromise. UA PI > 95th percentile is considered an abnormal range (value more than 1.2). MCA PI<5th percentile was considered an abnormal range (value less than 0.9) [12]. Studies [13] comparing the diagnostic performance of fetal umbilical Doppler and MCA Doppler ultrasonography in suspected Fetal growth-restricted fetuses found that MCA Doppler indices are a better predictor for fetal outcome in terms of sensitivity and predictive value.

This abstract was previously presented as an oral paper at the 46th Annual Conference of the Association of Obstetricians and Gynecologists of Delhi, held from November 22 to 24, 2024.

## Materials and methods

This study was a prospective observational analysis conducted from July 2018 to May 2020 at a tertiary care center. It included 91 cases of growth-restricted fetuses. The participants were antenatal patients attending the Obstetrics and Gynecology clinic with clinical and sonographic evidence of FGR. FGR was defined as estimated fetal weight (EFW) or abdominal circumference (AC) below the third percentile, or the tenth percentile, accompanied by abnormal Doppler findings in the UA, MCA, or cerebroplacental ratio (CPR) [14-18]. After delivery, 91 cases were divided into two groups: cases with small for gestational age (SGA) fetuses (birth weight less than 10th percentile for gestational age) and non-SGA cases (birth weight ≥ 10th percentile for gestational age) based on the Hadlock formula [19]. The gestational age of included patients ranged between 28 and 38 + 6 weeks, and only singleton pregnancies were considered. Exclusion criteria encompassed multiple gestations, fetuses without growth restriction, congenital malformations, or chromosomal abnormalities. The study was done after taking ethical clearance and informed consent from the participants. Doppler ultrasound evaluations were performed using the Philips Affiniti 30 ultrasound machine (Philips Healthcare, Andover, MA) equipped with a 2-6 MHz curvilinear probe. The UA was assessed by identifying a free-floating loop of the umbilical cord and optimizing the angle of insonation. Abnormal UA Doppler indices were defined as an S/D ratio ≥3 or PI values above the 95th percentile. The MCA was examined at its proximal segment near the greater wing of the sphenoid, with an insonation angle of 0-15 degrees. Doppler indices, including the S/D ratio, resistance index (RI), and PI, were calculated for both UA and MCA. Statistical analysis was performed using SPSS software (version 21.0; IBM Corp., Armonk, NY). Quantitative variables were compared using either the Independent t-test or the Mann-Whitney test, depending on data distribution. Qualitative variables were analyzed using the chi-square or Fisher’s exact test. Diagnostic tests were employed to calculate the sensitivity, specificity, positive predictive value (PPV), and negative predictive value (NPV) of the PI of the MCA, S/D ratio of the UA, and PI of the UA for predicting adverse perinatal outcomes. Comparisons of sensitivity and specificity were made using the McNemar test, with a *P*-value < 0.05 considered statistically significant. The clinical management of patients was tailored based on their overall condition and Doppler findings, with pregnancy termination undertaken when indicated to optimize perinatal outcomes. Data analysis was completed using Microsoft Excel for data entry and SPSS for statistical computations.

## Results

The study included 91 FGR pregnancies, which were divided into two groups after delivery: 69 cases classified as SGA and 22 cases as non-SGA. The clinical characteristics of the participants are given in Table 1. Amniotic fluid index (AFI) analysis revealed that 55.07% of SGA cases had normal AFI, while 44.93% were diagnosed with oligohydramnios, showing a significant statistical difference (*P* = 0.011). The mean maternal age was 28.77 ± 4.79 years for SGA cases and 27.5 ± 3.8 years for non-SGA cases. The mean birth weight for SGA cases was 1.85 ± 0.49 kg, significantly lower than the 2.53 ± 0.23 kg observed in non-SGA cases. Forty-nine neonates (71.01%) had a birth weight below the 3rd percentile, while 20 cases (28.99%) fell within the 3rd to 10th percentile (*P* < 0.001).

**Table 1:** Clinical characteristics of the study subjects. SGA, small for gestational age; BMI, basal metabolic index; IHCP, intrahepatic cholestasis of pregnancy; HTN, hypertension

Variables	SGA (*n* = 69)	Non-SGA (*n* = 22)	*P*-value	Test performed
Age (years)	28.77 ± 4.79	27.5 ± 3.8	0.261	t Test
BMI	23.99 ± 4.61	23.81 ± 2.91	0.946	Fisher's exact test
Parity				
Primi	31 (44.91%)	11 (50%)	0.678	Chi-square test
Multi	38 (55.07%)	11 (50%)		
Risk factors				
Pre-eclampsia	10 (14.49%)	0	0.111	Fisher's exact test
Gestational HTN	9 (13.04%)	2 (9.09%)		
Overt diabetes	1 (1.45%)	0		
Gestational diabetes	15 (21.74%)	6 (27.27%)		
IHCP	7 (10.14%)	5 (22.73%)		
Anemia	3 (4.35%)	0		
Preterm	29 (43.48%)	7 (31.82%)		
Others	30 (43.48%)	13 (59.09%)		
Oligohydramnios				
No	38 (55.07%)	19 (86.36%)	0.011	Fisher’s exact test
Yes	31 (44.93%)	3 (13.64%)		

The mode of delivery varied between the groups, with 59.42% of SGA cases undergoing cesarean section compared to 36.36% in the non-SGA group; however, this difference was not statistically significant (*P* = 0.059). Perinatal outcomes for both groups are presented in Table 2. The Appearance, Pulse, Grimace, Activity, and Respiration (APGAR) scores at 5 minutes showed no significant difference between the groups (*P* = 0.331), with only 7.25% of SGA neonates scoring ≤7, while 92.75% scored >7. However, Neonatal Intensive Care Unit (NICU) admissions were significantly higher among SGA neonates, affecting 70.15% compared to 31.82% of non-SGA neonates (*P* = 0.001). The mean duration of NICU stay was also significantly longer for SGA neonates (9.15 ± 12.94 days) than for non-SGA neonates (2.5 ± 1.05 days, *P* = 0.018). Neonatal complications were more prevalent in the SGA group, affecting 61.76% of cases, compared to 22.73% in the non-SGA group, a statistically significant difference (*P* = 0.001). These findings highlighted the significant differences in perinatal outcomes between SGA and non-SGA pregnancies, underscoring the utility of Doppler indices in predicting perinatal complications.

**Table 2:** Perinatal outcomes. NICU, Neonatal Intensive Care Unit; PI, pulsatility index; S/D, systolic/diastolic ratio; APGAR, Appearance, Pulse, Grimace, Activity, and Respiration

Variables	SGA (*n* = 69)	Non-SGA (*n* = 22)	*P*-value	Test performed
Type of delivery				
Cesarean section	41 (59.42%)	8 (36.36%)	0.059	Chi-square test
Vaginal delivery	28 (40.58%)	14 (63.64%)		
Birth weight percentile				
<3rd	49 (71.01%)	0 (0%)	<0.0001	Fisher's exact test
3rd-10th	20 (28.99%)	0 (0%)		
>10th	0 (0%)	22 (100%)		
Mean birth weight (kg ± SD)	1.85 ± 0.49	2.53 ± 0.23	<0.0001	t-test
5th minute APGAR score ≤7	5 (7.25%)	0	0.331	Fisher's exact test
NICU admission	47 (70.15%)	7(31.82%)	0.001	Chi-square test
Days of admission (mean ± SD)	9.15 ± 12.94	2.5 ± 1.05	0.018	Mann-Whitney test
Neonatal complications				
Yes	42 (61.76%)	5 (22.73%)	0.001	Chi-square test
No	26 (38.24%)	17 (77.27%)		
Adverse perinatal outcome				
Yes	69 (100%)	12 (54.55%)	<0.0001	Fisher's exact test
No	0	10 (45.45%)		
Middle cerebral artery PI	1.22 ± 0.5	1.48 ± 0.49	0.017	Mann-Whitney test
Umbilical artery PI	1.05 ± 0.41	0.97 ± 0.33	0.571	Mann-Whitney test
Umbilical artery S/D	3.26 ± 1.08	2.8 ± 0.84	0.023	Mann-Whitney test

The association between Doppler indices of the UA and MCA with various perinatal outcomes is shown in Tables 3-5. In Table 3, abnormal UA S/D ratios were associated with higher rates of cesarean delivery (71.05% vs. 40.82%, *P* = 0.005), birth weight <10th percentile (86.84% vs. 65.31%, *P* = 0.022), NICU admissions (78.38% vs. 44.90%, *P* = 0.002), and neonatal complications (65.79% vs. 38.78%, *P* = 0.012). Table 4 shows that abnormal UA P/I was linked to higher rates of neonatal complications (68.18% vs. 43.94%, *P* = 0.04). While other perinatal outcomes such as cesarean delivery (68.18% vs. 48.48%, *P* = 0.109), NICU admissions (76.19% vs. 53.85%, *P* = 0.07), and NICU stay >48 hours (81.25% vs. 70.59%, *P* = 0.508) showed higher percentages in the abnormal group, these differences were not statistically significant. In Table 5, abnormal MCA PI showed a significant association with birth weight <10th percentile (89.66% vs. 69.35%, *P* = 0.039).

**Table 3:** Association between perinatal outcomes and umbilical artery S/D ratio. NICU, Neonatal Intensive Care Unit; S/D, systolic/diastolic ratio; APGAR, Appearance, Pulse, Grimace, Activity, and Respiration

Perinatal outcome	Abnormal Doppler (*n *= 42)	Normal Doppler (*n* = 49)	Total (*n *= 91)	*P*-value	Test performed
Cesarean section	27 (71.05%)	20 (40.82%)	47 (54.02%)	0.005	Chi-square test
APGAR ≤ 7	3 (7.89%)	1 (2.04%)	4 (4.60%)	0.314	Fisher's exact test
Birth weight < 10th percentile	33 (86.84%)	32 (65.31%)	65 (74.71%)	0.022	Chi-square test
NICU admission	29 (78.38%)	22 (44.90%)	51 (59.30%)	0.002	Chi-square test
NICU stay (>48 hours)	22 (75.86%)	15 (71.43%)	37 (74%)	0.724	Chi-square test
Neonatal complications	25 (65.79%)	19 (38.78%)	44 (50.57%)	0.012	Chi-square test
Perinatal mortality	1 (2.63%)	0 (0%)	1 (1.15%)	0.437	Fisher's exact test

**Table 4:** Association between perinatal outcome and umbilical artery PI. NICU, Neonatal Intensive Care Unit; PI, pulsatility index; APGAR, Appearance, Pulse, Grimace, Activity, and Respiration

Perinatal outcome	Abnormal Doppler (*n* = 25)	Normal Doppler (*n* = 66)	Total (*n *= 91)	*P*-value	Test performed
Cesarean section	15 (68.18%)	32 (48.48%)	47 (53.41%)	0.109	Chi-square test
APGAR ≤ 7	2 (9.09%)	3 (4.55%)	5 (5.68%)	0.595	Fisher's exact test
Birth weight < 10th percentile	17 (77.27%)	49 (74.24%)	66 (75%)	0.776	Chi-square test
NICU admission	16 (76.19%)	35 (53.85%)	51 (59.30%)	0.07	Chi-square test
NICU stay (>48 hours)	13 (81.25%)	24 (70.58%)	37 (74%)	0.508	Fisher's exact test
Neonatal complications	15 (68.18%)	29 (43.94%)	44 (50%)	0.049	Chi-square test
Perinatal mortality	1 (4.55%)	1 (1.52%)	2 (2.27%)	0.44	Fisher's exact test

**Table 5:** Association between perinatal outcome and middle cerebral artery PI. NICU, Neonatal Intensive Care Unit; PI, pulsatility index; APGAR, Appearance, Pulse, Grimace, Activity, and Respiration

Perinatal outcome	Abnormal Doppler (*n *= 29)	Normal Doppler (*n *= 62)	Total	*P*-value	Test performed
Cesarean section	16 (55.17%)	33 (53.23%)	49 (53.85%)	0.862	Chi-square test
APGAR <= 7	1 (3.45%)	4 (6.45%)	5 (5.49%)	1	Fisher's exact test
Birth weight <10th percentile	26 (89.66%)	43 (69.35%)	69 (75.82%)	0.039	Fisher's exact test
NICU admission	19 (67.86%)	35 (57.38%)	54 (60.67%)	0.347	Chi-square test
NICU stay > 48 hours	16 (84.21%)	24 (70.59%)	40 (75.47%)	0.334	Fisher's exact test
Neonatal complications	17 (58.62%)	30 (48.39%)	47 (51.65%)	0.363	Chi-square test
Perinatal mortality	1 (3.45%)	1 (1.61%)	2 (2.20%)	0.538	Fisher's exact test

**Figure 1:**
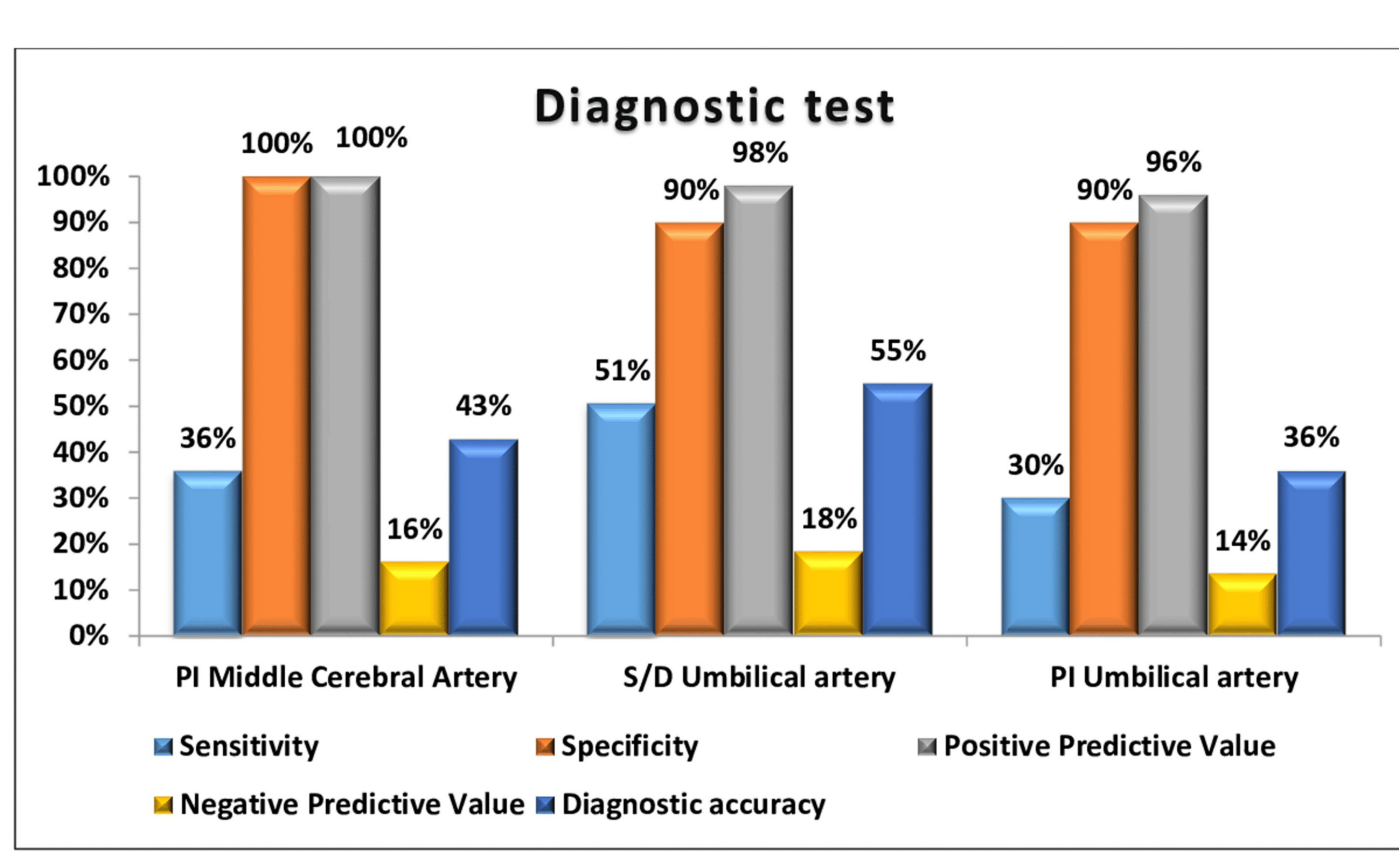
Sensitivity, specificity, PPV, and NPV of middle cerebral artery PI, umbilical artery S/D ratio, and umbilical artery PI for predicting adverse perinatal outcome PI, pulsatility index; S/D, systolic/diastolic ratio; NPV, negative predictive value; PPV, positive predictive value

Figure 1 illustrates the predictive value of Doppler indices for adverse perinatal outcomes. Among the indices, the UA S/D ratio demonstrated the highest diagnostic accuracy (54.95%), with a sensitivity of 50.62% and a specificity of 90%. It also showed a strong PPV of 97.62%, but a lower NPV of 18.37%. The MCA PI showed the highest specificity and PPV at 100%, indicating its reliability for confirming adverse outcomes, though its sensitivity was only 35.8%, limiting its ability to detect all cases. The UA PI had a specificity of 90% and PPV of 96%, with a sensitivity of 29.63% and diagnostic accuracy of 36.26%. These findings underscore the need to use these indices together for a more comprehensive assessment of adverse perinatal outcomes.

## Discussion

FGR poses significant challenges in obstetric care, and accurate prediction of adverse perinatal outcomes is critical for timely intervention. This study compared the utility of Doppler indices of the MCA and UA in predicting perinatal outcomes in pregnancies affected by FGR, providing valuable insights into their clinical applicability.

The findings indicate that abnormal Doppler indices, particularly of the UA S/D ratio, were strongly associated with adverse perinatal outcomes, including cesarean delivery, NICU admission, longer NICU stay, and neonatal complications. Among the 91 participants, the SGA group exhibited significantly poorer outcomes compared to the non-SGA group, underscoring the importance of Doppler ultrasonography in identifying high-risk pregnancies.

The SGA group had significantly lower mean birth weights (1.85 ± 0.49 kg) compared to the non-SGA group (2.53 ± 0.23 kg, *P* < 0.0001), consistent with the diagnosis of growth restriction. NICU admissions were significantly more common in the SGA group (70.15% vs. 31.82%, *P* = 0.001), with a longer mean NICU stay (9.15 ± 12.94 days vs. 2.5 ± 1.05 days, *P* = 0.018). These findings highlight the heightened vulnerability of SGA neonates to complications, necessitating closer monitoring and timely delivery to mitigate risks. Brar et al. [20] noted that when the UA S/D ratio was >3, there was a higher likelihood of SGA, a 5-minute APGAR score <7, and cesarean section, which correlates with the findings of the present study.

Neonatal complications, including respiratory distress syndrome, sepsis, and metabolic disturbances, were more prevalent in the SGA group (61.76% vs. 22.73%, *P* = 0.001). This aligns with existing literature, emphasizing the increased morbidity associated with FGR, often stemming from placental insufficiency and hypoxia [12]. The significant correlation between abnormal Doppler indices and these outcomes reinforces their value in clinical decision-making.

The UA S/D ratio emerged as the most reliable predictor of adverse perinatal outcomes, with the highest diagnostic accuracy (54.95%). Its sensitivity (50.62%) and specificity (90%) suggest that it is effective in identifying high-risk pregnancies, although the limited NPV (18.37%) indicates that some at-risk fetuses may be missed. The strong PPV (97.62%) underscores its utility in confirming adverse outcomes when Doppler findings are abnormal.

The MCA PI demonstrated 100% specificity and PPV, making it a robust tool for ruling out adverse outcomes. However, its low sensitivity (35.8%) highlights its limitations in detecting all cases of FGR. This is consistent with previous studies suggesting that MCA vasodilation, a compensatory mechanism in FGR, occurs relatively late in the disease process and may not always be apparent in early-stage growth restriction.

The UA PI showed moderate performance, with a diagnostic accuracy of 36.26%, specificity of 90%, and sensitivity of 29.63%. While its PPV (96%) was high, its lower sensitivity and diagnostic accuracy suggest it may be more effective as a complementary tool rather than a standalone predictor. Our findings were consistent with other studies, including those by Dhand et al. [21] and Gramellini et al. [22]. However, the findings of Sachin et al. [23] were not comparable to those of the present study.

Abnormal UA S/D ratios were significantly associated with increased rates of cesarean delivery (71.05% vs. 40.82%, *P* = 0.005), birth weight below the 10th percentile (86.84% vs. 65.31%, *P* = 0.022), NICU admissions (78.38% vs. 44.90%, *P* = 0.002), and neonatal complications (65.79% vs. 38.78%, *P* = 0.012). These findings were consistent with other studies done in the southern part of the Indian subcontinent [24]. These findings were consistent with the pathophysiology of FGR, where elevated UA resistance reflects placental insufficiency and impaired oxygen exchange.

The findings of this study have important clinical implications. The high specificity and PPV of MCA PI and UA S/D ratio make them valuable tools for confirming adverse outcomes, enabling timely interventions such as expedited delivery or enhanced neonatal care. However, their relatively low sensitivity underscores the need for a multifaceted approach, combining Doppler indices with other clinical and biochemical markers to improve the identification of at-risk pregnancies.

The significant association of abnormal Doppler findings with NICU admissions, longer NICU stays, and neonatal complications highlights the role of these indices in guiding perinatal management. For instance, serial Doppler assessments can help monitor disease progression, optimize the timing of delivery, and reduce the risk of stillbirth or severe neonatal morbidity.

This study contributes to the growing body of evidence supporting the use of Doppler ultrasonography in managing FGR. Its strengths include a well-defined cohort, robust statistical analysis, and detailed evaluation of multiple Doppler indices. However, some limitations must be acknowledged. The sample size was relatively small, which may limit the generalizability of the findings. 

Future research should focus on integrating Doppler findings with novel biomarkers and advanced imaging techniques to improve the sensitivity and diagnostic accuracy of FGR prediction. Larger prospective studies are also needed to validate these findings and explore the long-term implications of abnormal Doppler indices on neurodevelopmental and metabolic outcomes.

## Conclusions

This study highlights the critical role of Doppler ultrasonography in predicting adverse perinatal outcomes in FGR. The UA S/D ratio demonstrated the highest diagnostic accuracy, while the MCA PI provided exceptional specificity and PPV. Together, these indices offer valuable insights into the severity of growth restriction and guide perinatal management strategies. A comprehensive approach incorporating Doppler findings along with clinical and biochemical markers is essential to optimize outcomes for both mother and fetus.
